# Usefulness of Published PCR Primers in Detecting Human Rhinovirus Infection

**DOI:** 10.3201/eid1702.101123

**Published:** 2011-02

**Authors:** Cassandra E. Faux, Katherine E. Arden, Stephen B. Lambert, Michael D. Nissen, Terry M. Nolan, Anne B. Chang, Theo P. Sloots, Ian M. Mackay

**Affiliations:** Author affiliations: The University of Queensland, Brisbane, Queensland, Australia (C.E. Faux, K.E. Arden, S.B. Lambert, M.D. Nissen, T.P. Sloots, I.M. Mackay);; The University of Melbourne, Melbourne, Victoria, Australia (T. Nolan);; Royal Children’s Hospital, Brisbane (A.B. Chang)

**Keywords:** Rhinovirus, respiratory infection, polymerase chain reaction, diagnosis, genotype, comparative study, viruses, dispatch

## Abstract

We conducted a preliminary comparison of the relative sensitivity of a cross-section of published human rhinovirus (HRV)–specific PCR primer pairs, varying the oligonucleotides and annealing temperature. None of the pairs could detect all HRVs in 2 panels of genotyped clinical specimens; >1 PCR is required for accurate description of HRV epidemiology.

Human rhinoviruses (HRVs) cause more asthma exacerbations than any other known factor, in addition to causing most colds and influenza-like illnesses. The prevalence of HRV in published reports varies considerably. A novel HRV clade identified in 2006, now known as HRV species C (HRV-C) (*1*), can be identified only by PCR. Since 1988, seasonality and clinical outcomes and numerous different primer pairs have been used to identify HRV; how well these methods perform on new HRV types is uncertain. Given the likely variation in the preparation of RNA, the quality and formulations of commercial reverse transcription (RT)-PCR enzymes and reaction mix components and changes in thermal cyclers since 1988, not surprisingly many, perhaps most, of these assays are not being used in the manner they were originally described. For example, the first HRV-specific primers reported (*2*) have subsequently been used with different RNA preparation methods, amounts of reverse transcriptase, cDNA priming strategies, dNTP concentrations, annealing temperatures (T_M_s), and cycling conditions (*3**,**4*).

## The Study

We conducted a preliminary comparison of the relative sensitivity of a cross-section of published HRV-specific PCR primer pairs (most of which were first published before HRV-C was reported), independent of most variables described above, by testing a panel of 57 clinical specimen nucleic acid extracts from combined nose and throat swabs from preschool children with colds and influenza-like illnesses in Melbourne, Australia. The study was approved by the Royal Children’s Hospital Human Research Ethics Committee. The panel included representatives of the 3 HRV species (Figure), human enteroviruses (HEVs), and extracts negative for picornaviruses. The HRVs had been previously detected by using a nested primer pair (Table A1) (*5*). We used 10 different HRV primer pairs and also retested specimens by using the original primer pair with our standard reagents and equipment (*5*). We applied the published T_M_ when possible. The original descriptions of primer pairs 7 and 10 (Table A1) lacked T_M_ information, and after in-house calculations, we used T_M_s of 50°C and 58°C, respectively. We also deliberately standardized the reagents (OneStep RT-PCR kit, QIAGEN, Doncaster, Victoria, Australia) and thermal cyclers used (Veriti, Applied Biosystems, Foster City, CA, USA) for conventional PCR and the RotorGene 3000 real-time cycler (QIAGEN). Because primer pair 1 had a published history of detecting types from all HRV species, we chose it to genotype HRV-positive samples by sequencing the amplified products. Other pairs were used if pair 1 was unsuccessful.

**Figure:**
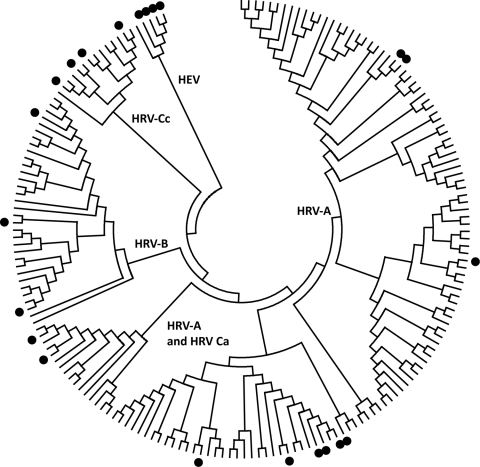
Distribution of human rhinovirus (HRV) and human enterovirus (HEV) sequences used for primer pair studies. The HRV and HEV genotypes from the testing panel (indicated by filled circles) were aligned with the central 154 nt of the 5′ untranslated region (UTR) region of all complete HRV genomes and poliovirus-1. HRV-Ca and HRV-Cc refer to HRV-Cs with 5′ UTR sequences that have phylogenetic origins from either HRV-As or HRV-Cs, respectively. The tree was constructed by neighbor joining of maximum composite likelihood distance implemented in MEGA (www.megasoftware.net).

We found that no primer pair detected the same HRVs and HEVs typed when the original pair (*5*) or pair 1 (Table A1) was used. Five primer pairs, including real-time PCR (rtPCR) pair 5, did not amplify the HEVs, a positive feature for HRV-specific studies. Only 2 primer pairs amplified anything from a specimen that was positive for both HRV and HEV, a problem for accurate estimation of the frequency of co-detections. The original primer pair screen detected 3 untypeable picornaviruses, which were not detected by any other pair or by repeat testing using the same pair. Only the second-round amplicon of the 3 nested sets of nested primer pairs (*2*,*3, and*,*9*) was considered because the second round increased the total number of positive specimens over the first round. The longest amplicon, produced by primer pair 7, was also a valuable genotyping target, but it detected only 14 of the original 27 HRV-positive specimens in this population.

We next selected 4 frequently published primer pairs (*1*,*5*,*7, and*,*8*) to examine 44 picornavirus-positive specimens (39 HRVs, 3 HEVs, and 2 untypeable picornaviruses) from nonhospitalized children with acute asthma exacerbation (*6*). As before, primer pair 1 detected the greatest number of HRV- and HEV- positive specimens and all positive specimens detected by other primer sets (n = 41), followed by pair 7 (n = 40), pair 5 (n = 36), and pair 8 (n = 31). Most notably, primer pair 7 performed better than it had in the previous population, detecting only 1 fewer HRV than primer pair 1 and 9 more HRVs than pair 8. No species-specific bias was apparent, but generally, a specimen with a lower RNA concentration, as indicated by the cycle threshold from primer pair 5, was less likely to be detected or typed by using other primer pairs. Primer pairs 5 and 8 did not detect the 3 HEVs (HEV-68). We noted in both populations that primer pair 1 sometimes amplified a region of human genomic DNA from chromosome 6 (GQ497714), for which amplicon size was indistinguishable from that expected due to HRV.

It was not possible to use the precise conditions reported for the 10 compared assays; 1 was published >2 decades ago and used phenol chloroform extraction. Some of the original enzyme formulations or reagents are no longer available, and production processes have changed in the interim. Thermal cyclers have also changed. There was no consensus on enzymes and reaction mixes used. In addition, the previously published primers were used in assays divided between those using 1-step RT-PCR and those using a separate RT cDNA synthesis step. A review of studies that detected HRVs with adequately described conditions during 2009–2010 found that fewer used a single-tube RT-PCR approach than a 2-step system. We conducted single-tube RT-PCR to maintain the benefits of the so-called closed amplification system of rtPCR. Thus, we chose to use a single common set of reagents as the fairest way to compare the primer pairs examined in this study. We believe the nature of this relative comparison best reflects performance for the likely end users: clinical microbiology laboratories or researchers.

We compared primers rather than assay function using clinical material instead of cultured virus, plasmid or synthetic RNA standards, or screening contemporary or archived extracts, which are sometimes of low viral load. When picornavirus epidemiology is the primary research focus, we recommend using >2 primer pairs to maximize the detection of HRVs. Under our conditions, pairs 1–4 returned the highest number of positive results, and the rtPCRs behaved similarly but with reduced sensitivity. The rtPCR that used pair 5 did not amplify known HEVs.

Many possible reasons could cause discrepant virus testing results between different sites, including changes to specimen integrity resulting from transport and variable amplification resulting from low viral loads. The effects of viral load can be seen in this study: specimens in population 1 that were positive with multiple (>6 separate pairs) primer pairs had a mean cycle threshold of 33.3 (combining results from both rtPCRs), whereas those with <6 positive results had means of 39.3 cycles. Most (29/33) specimens with <3 positive primer pairs were negative by rtPCR. Amplification variability can also be attributed to the substantial nucleotide sequence diversity between HRVs and the different temporal and clinical characteristics of the 2 specimen populations we used. Population diversity is a feature of HRV studies in the literature.

## Conclusions

Our selection of published primer pairs includes those from studies that have informed our current understanding of HRV epidemiology. Finding such a high degree of variability in performance was thus noteworthy. Inefficient HRV detection by PCR may be a serious problem for research studies. Comparison of data between different HRV studies is confounded as are data from studies seeking to determine the effects of other respiratory viruses. The prevalence, seasonality, transmission, and clinical effects of HRV types and species require reexamination with tools that have been comparatively validated to ensure their sensitivity.

## Appendix

**Table A1:** Results from RT-PCR with 10 different primer pairs, targeting HRVs by using a panel of 57 clinical nucleic acid extracts from combined nose and throat swabs, Melbourne, Australia

Specimen no.	Species	HRV identifier†	Primer pairs										
			Original‡ (5)	1§ (7)	2 (8)	3 (5)	4 (9)	5¶ (10)	6 (11)	7 (12)	8 (13)	9 (14)	10 (15)
1	HRV-A	HGD and MC1202	+	+	+	+	+	39.1	–	–	+	–	–
2	HRV-C	PUMCH2516	–	–	–	–	+	–	–	–	–	–	–
3	HRV-A	MC1202	+	+	+	+	+	33.9	32.7	+	+	–	+
4	HRV-A	HRV-78	+	+	-	+	+	37.8	36.2	–	–	+	–
5	HRV-A	HRV-80	–	+	+	+	+	35.1	33.2	+	–	+	+
6	HRV-A	HRV-54	–	+	+	+	+	38.1	40.3	–	+	–	+
7	HRV-B	HRV-92	–	+	+	+	+	32.0	30.7	+	+	+	–
8	HRV-B	PUMCH2910	+	+	+	+	+	34.9	32.2	–	+	–	–
9	HRV-B	HRV-6#	–	+	+	–	–	–	–	–	+	–	–
10	HRV-B	PUMCH5056	–	+	+	+	+	29.4	27.5	+	+	+	–
11	HRV-B	HRV-6	+	-	+	+	+	40.0	39.9	–	–	–	–
12	HRV-C	NH363	+	+	+	+	+	38.3	39.0	+	+	–	–
13	HRV-C	CL-170085	+	+	+	+	+	35.7	32.6	+	–	+	+
14	HRV-C	N33	+	+	+	+	+	35.2	32.49	+	–	+	+
15	HRV-C	W11	+	+	+	+	+	–	–	–	–	–	–
16	HRV-C	A98128507	+	+	+	+	+	–	–	+	+	–	–
17	HRV-C	A99038140	+	+	+	+	+	37.8	33.5	–	+	+	+
18	HRV-C	HRV-N10	+	+	+	+	–	–	–	–	–	–	–
19	HRV-C	HRV-QCE**	–	+	-	+	+	35.7	–	–	+	–	–
20	HRV-C	W18	+	+	+	–	–	–	–	–	–	–	–
21	HRV-C	N42	+	+	+	+	+	41.1	34.7	+	+	–	–
22	HRV-C	CL-1237693	+	+	-	–	–	–	–	–	–	–	–
23	HRV-C	NH4443	+	+	+	+	+	39.8	–	+	+	–	–
24	HRV-C	HRV-QCE	–	+	+	+	+	33.8	23.9	+	+	+	+
25	HRV-C	W30	+	+	–	+	+	–	37.9	–	–	–	–
26	HRV-C	KR2031	–	–	+	+	-	–	–	–	–	–	–
27	HRV-C	SO4302	–	+	+	+	+	30.1	28.6	+	+	+	–
28	HRV-C	RV1051	–	+	+	+	+	31.3	29.9	+	+	+	+
29	HRV-C	N46	+	+	+	+	+	23.7	18.9	+	+	+	+
30	HEV-B	HEV-97	+	+	–	–	–	–	–	–	–	–	–
31	HEV-B	CV-B3	–	+	–	+	+	–	38.2	–	–	–	–
32	HEV-A/ HRV-C	CV-A16 and W18††	+	+‡‡	-§§	–	–	–	–	–	+	–	–
33	PV	UT	–	–	–	–	+	–	–	–	–	–	–
34	PV	UT	+	–	+	+	-	–	38.8	–	–	–	–
35	PV	UT	+	–	–	+	+	–	41.7	–	–	–	–
36	PV	UT	–	–	+	–	–	–	–	–	–	–	–
37	PV	UT	–	–	–	–	–	39.6	37.6	–	–	–	–
38	PV	UT	+	–	+	–	–	–	–	–	–	–	–
39–41	PV	NA	+	–	–	–	–	–	–	–	–	–	–
42–56	Neg	NA	–	–	–	–	–	–	–	–	–	–	–
57	Neg	NA	–	–	–	–	–	–	43.4	–	–	–	–
Total positive			26	29	27	28	27	21	23	14	18	11	9
% of all specimens			46	51	47	49	47	37	40	25	32	19	16

*RT-PCR, reverse transcription PCR; HRV, human rhinovirus; HEV, human enter0virus, HGD, human genomic DNA; PV, member of the family *Picornaviridae* of unknown type; CV, coxsackievirus; UT, untypeable; neg, negative; NA, typing not attempted because there was no PCR product; +, detected; –, not detected, †Based on best match returned by comparison of the clinical specimen's HRV sequence, derived from the amplicon, to the GenBank database. ‡Primer pair originally used and described elsewhere (*1*) to screen these specimens. Repeated here as primer pair 3. §Amplicon from these primers was most commonly used to identify the HRV type and to assign a species. ¶This real-time RT-PCR was used to test the extracts during evaluation and at the end of the evaluation; steady threshold cycle values indicated that, for 5′ UTR at least, the extracted RNA had not deteriorated during the testing process. Numbers are cycle threshold values. #Sequence identity suggested a novel HRV-B type. **Sequence identity suggested a novel HRV-C type. ††Two distinct virus sequences determined only after amplicon cloning and direct bacterial colony PCR screening and nucleotide sequencing. ‡‡Undiscriminated positive before cloning. §§Indicates negative for either virus.
